# Supplementary material for: Low Cell Number Proteomic Analysis Using In-Cell Protease Digests Reveals a Robust Signature for Cell Cycle State Classification
Source: Mol Cell Proteomics. 2021 Nov 4;21(1):100169. doi: 10.1016/j.mcpro.2021.100169 (PMC8760417; doi:10.1016/j.mcpro.2021.100169)
Supplement: Supplemental Figures S1–S3 [file mmc8.pdf]

Supplementary data for

Low cell number proteomic analysis using in-cell protease digests reveals a robust signature for cell cycle state classification

Van Kelly[1,2], Aymen al-Rawi[1], David Lewis[1], Georg Kustatscher[1,2] and Tony Ly[1,3]*

Contents:
Supplementary Figure S1
Supplementary Figure S2
Supplementary Figure S3

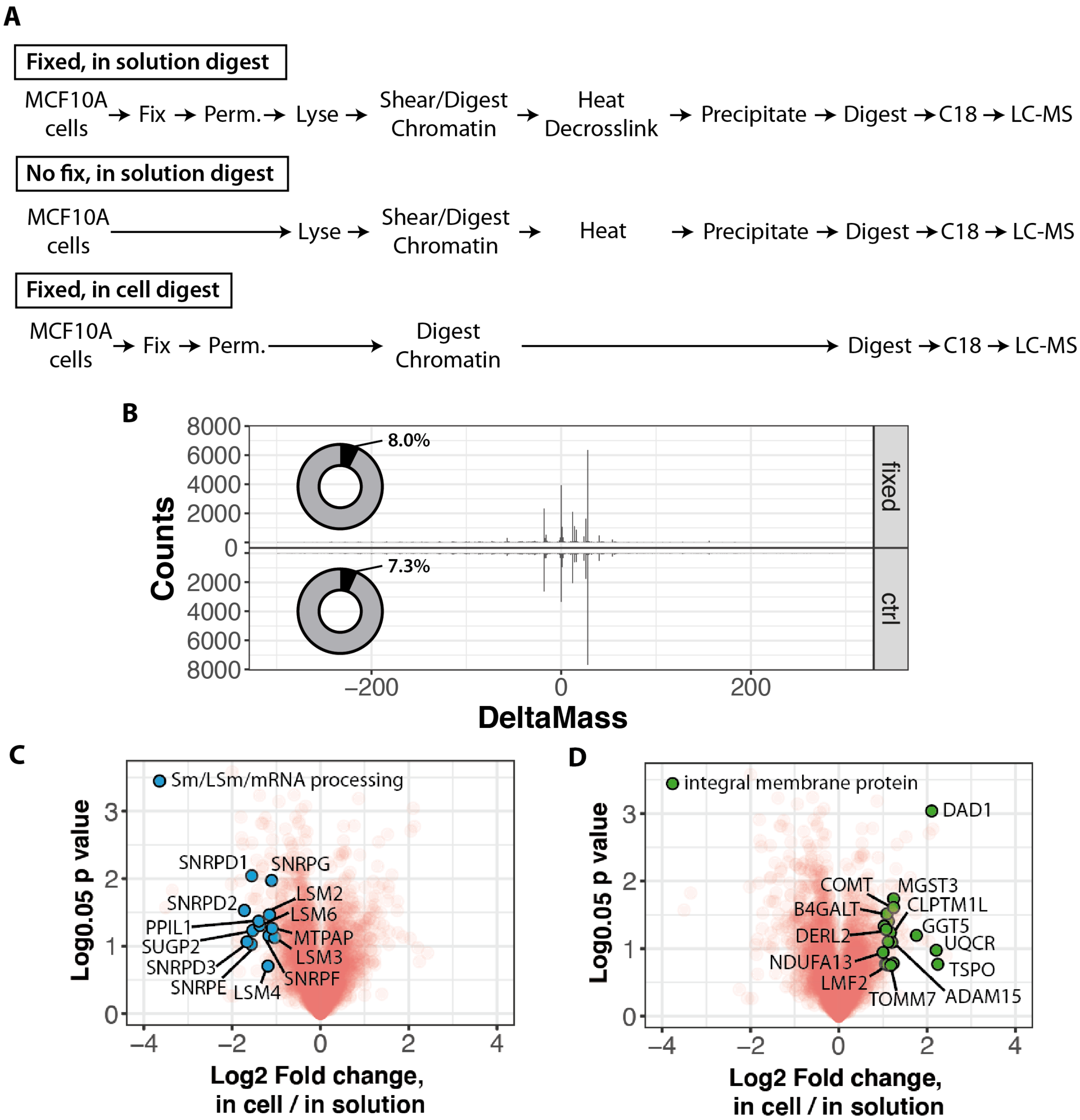

Supplementary Figure S1. A) Scheme in Fig. 1A expanded to include details. B) Results from an error-tolerant dependent peptide search (MaxQuant) comparing control (cells without fixative) and fixed cells. C,D) Volcano plots comparing triplicate in cell and in solution digests.

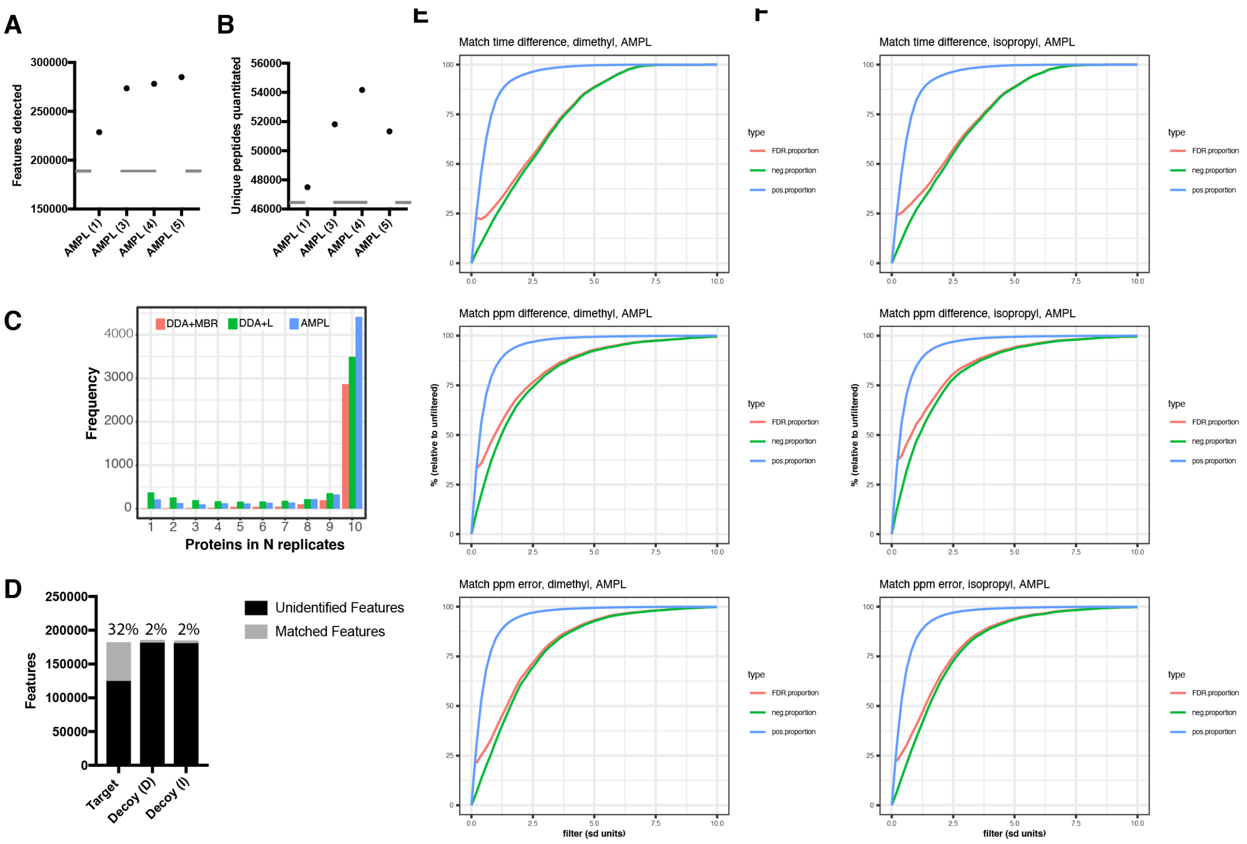

Supplementary Figure S2. A) Identified features in target and decoy proteomes, including proportion of features matched to library. B, C) Optimising the number of MS1 spectra to average by measuring impact on the number of features and peptides detected. D) An analysis of data completeness comparing DDA, DDA with a library (DDA+L) and MPL. E, F) ROC plots for the dimethyl (E) and isopropyl (F) decoy proteomes filtering against match time difference, match ppm difference and match ppm error. Proportions are given in %s. FDR proportion is the estimated FDR at indicated filtering threshold over FDR with no filter.

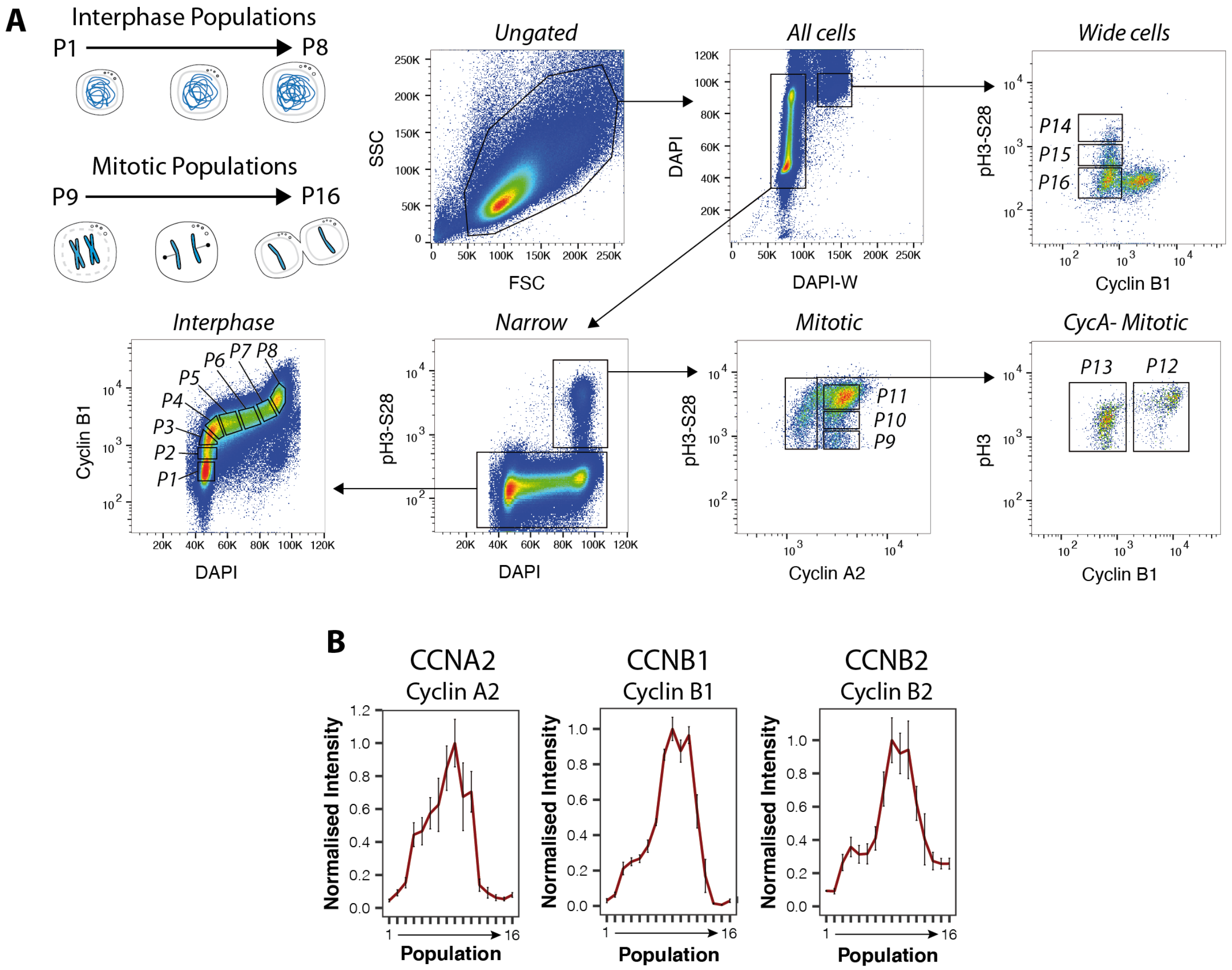

Supplementary Figure S3. A) Scheme of FACS isolation and pseudocolour plots showing the gating strategy to isolate the 16 cell cycle populations. B) Mean normalised intensities across the 16 cell cycle populations as measured by MS for cyclin A2, cyclin B1 and cyclin B2.
